# Cadmium affects microtubule organization and post-translational modifications of tubulin in seedlings of soybean (*Glycine max* L.)

**DOI:** 10.3389/fpls.2015.00937

**Published:** 2015-11-06

**Authors:** Jarosław Gzyl, Jagna Chmielowska-Bąk, Roman Przymusiński, Edward A. Gwóźdź

**Affiliations:** Department of Plant Ecophysiology, Institute of Experimental Biology, Faculty of Biology, Adam Mickiewicz UniversityPoznań, Poland

**Keywords:** root, cadmium, microtubule cytoskeleton, immunocytochemistry, gene expression, immunoblotting, tubulin isoforms, post-translation modifications

## Abstract

Cadmium (Cd) is a non-essential heavy metal, toxic to all living organisms. The microtubule (MT) cytoskeleton appears to be one of the main targets of Cd action. In this study we present, with the use of various immunological approaches, the effect of Cd at moderate (85 μM) and high (170 μM) concentrations on the structure and functioning of the MT cytoskeleton in the root cells of soybean seedlings. As the result of heavy metal action, root growth was significantly diminished and was accompanied by a reduction in mitotic activity and disturbance in the structure of the MT arrays, including randomization of the cortical MT arrangement, distorted mitotic arrays and complete depolymerization of the MTs. Biochemical analysis revealed decreased levels of various α- and β-tubulin isoforms with a parallel down-regulation of most examined α-tubulin genes. Simultaneously, Cd treatment led to differentiated changes in the level of tubulin post-translational modifications, including tyrosination, detyrosination, acetylation, and polyglutamylation. Decreased tyrosination and polyglutamylation of particular tubulin isoforms accompanied by increase in the level of specific detyrosinated and acetylated isoforms implies augmented stability and reduced turnover of the MTs during stress conditions. Taken together, the obtained results indicate the significant impact of Cd on gene expression levels and subsequent post-translational processing of tubulin, which may be related to the impairment of MT cytoskeleton functioning in root cells.

## Introduction

About 25,000 t of cadmium (Cd) per year is released into the environment, mainly through weathering of rocks, forest fires, volcanoes, and human activity, such as mining, agriculture, sewage processing, automobiles, and the metal industry (Dalcorso et al., 2013; Tran and Popova, 2013). Therefore, Cd has become a widespread non-essential heavy metal and one of the most toxic and dangerous environmental pollutants, with relative high mobility in the soil-plant system and ability to interfere with plant metabolism. Moreover, the metal accumulated in plant tissues might be introduced into the food chain, posing concerns for both animal and human nutrition. It is estimated that approximately 98% of ingested Cd comes from terrestrial foods (Tran and Popova, 2013).

The roots are well established as a main site of Cd action leading to numerous disorders, including inhibition of root growth, alterations in their morphogenesis (Rascio et al., 2008), interference with mitotic process by induction of chromosome aberrations (Siddiqui et al., 2009) and cytoskeleton dysfunction (Fusconi et al., 2007). Although microtubule (MT) cytoskeleton seems to be one of the main targets of Cd action (Přibyl et al., 2005; Xu et al., 2009; Malea et al., 2013), its functioning under heavy metal stress is not well elucidated. The MTs play crucial functions in the growth and development of plants and contribute to the highly ordered organization of the most important cellular processes, including formation of mitotic spindle, the cell plate, cell growth and elongation or intracellular transport and cell wall deposition (Horio and Murata, 2014). During the cell cycle, plant MTs are assembled into four distinct arrays: the interphase cortical MTs, pre-prophase band, mitotic spindle and phragmoplast. From a structural point of view, most MTs consist of 13 protofilaments which form a hollow cylinder. The protofilaments are polymers of heterodimers containing one α-tubulin and one β-tubulin subunit, each with a molecular weight of about 50 kDa (Parrotta et al., 2014). In spite of their quite simple structure, MTs can be precisely regulated throughout the cell cycle or cell differentiation to carry out diverse but specific cellular functions. The MT cytoskeleton may be controlled at a few different levels, and the final effect of MTs functioning is resultant of the expression of distinct tubulin genes (isotypes), tubulin post-translational modifications (PTMs) and a repertoire of MT-associated proteins (MAPs). The latter can regulate both the dynamics of MTs and their association with other cellular components as non-motor MAPs and MAPs with motor ability (Hamada, 2007). However, the primary level of tubulin heterogeneity is dependent on the differential expression of isotypes, which is probably regulated according to the specific developmental stage of a plant, the specific tissue or organ, as well as various internal and external stimuli (Oakley et al., 2007; Radchuk et al., 2007).

Plants have evolved a large heterogeneity in the number of α- and β-tubulins genes, and different plants possess diverse sets of tubulin genes (Parrotta et al., 2014). Presynthesised tubulin proteins can be next post-translationally modified to obtain differentiated subpopulations of MTs and to increase the heterogeneity of tubulin isoforms. The PTMs of tubulin are evolutionarily conserved and highly dynamic processes, therefore it is very likely that they play crucial functions in the eukaryotic cells (Parrotta et al., 2014). So far, several PTMs of tubulin have been recognized in plants, including tyrosination, detyrosination, acetylation (Smertenko et al., 1997b; Gilmer et al., 1999a,b; Nakagawa et al., 2013), phosphorylation (Blume et al., 2008; Ban et al., 2013), polyglutamylation (Wang et al., 2004), and transamidation (Del Duca et al., 2009). Most PTMs take place at the C-terminal tails of both tubulin subunits, and their level plays an essential role in the properties of MTs themselves as well as their interaction with associated proteins. Futhermore, PTMs may function individually and/or in combination to recruit specific protein complexes and thus govern the spatial and temporal regulation of the MT cytoskeleton during the cell cycle.

Tyrosination is a quite common and predominant modification because tubulins are normally synthesized with tyrosine as the last C-terminal amino acid. The enzymatic removal of C-terminal tyrosine by tubulin-specific carboxypeptidase generates detyrosinated tubulin (Glu-tubulin), which in turn can be subject to the opposite process of tyrosination with the use of specific enzyme tubulin tyrosine ligase (MacRae, 1997). It is assumed that the process of tubulin detyrosination is associated with increased MT stability, which is considered to protect MTs from depolymerisation (Peris et al., 2009). In acetylated tubulin isoforms, the acetyl group is attached to the α-amino group of lysine 40, and this PTM is detected in most cells in stable MTs (Piperno et al., 1987), but the exact function and significance of acetylated MTs in plants remain to be elucidated. Polyglutamylation is an abundant modification, which leads to the addition of glutamate side chains of variable length to the C-terminal tails of either α- or β-tubulin. In opposition to tyrosination, it is not processed on soluble tubulin subunits, but takes place, like detyrosination, on MTs (Parrotta et al., 2014).

Despite the importance of PTMs to proper MTs functioning, limited information is available concerning environmental stresses, including heavy metals treatment (Eleftheriou et al., 2013, 2015). In this report we documented the impact of Cd in moderate and high concentrations on the PTMs levels in the root tips of soybean. To our best knowledge this is the first report showing that Cd stress causes changes in the population of tyrosinated, detyrosinated, acetylated, and polyglutamylated isoforms of tubulin in plant cells. The proteomic approach based on a set of specific antibodies implies increased stability of MT fibers under stress conditions. Additionally, immunocytochemistry and ultrastructural observations proved clear impact of Cd on MTs functioning.

## Materials and Methods

### Plant Material, Growth Conditions and Treatment Procedures

Soybean seeds (*Glycine max* L. cv. Nawiko, kindly supplied by the Department of Genetics and Plant Breeding, University of Life Sciences, Poznań, Poland) were sterilized in 70% ethanol for 5 min and in 20% Clorox (1% sodium hypochlorite) for 10 min, rinsed with distilled water and imbibed for 4 h in distilled water (dH_2_O). The seeds were then germinated for 2 days in plastic dishes lined with filter paper moistened with dH_2_O. Seedlings with primary roots approximately 10 mm in length were transferred to Petri dishes (10 seedlings per dish) containing 4 ml of dH_2_O (control) or aqueous solutions of CdCl_2_^x^2.5H_2_O at different concentrations of the metal: 20, 80, 140, and 200 μM. The cultivation was carried out for 48 h in the dark at 22°C. Based on root measurements and tolerance index calculations (Wilkins, 1957) two concentrations of Cd were determined. As a result, the Cd concentration at which root growth was limited by approximately 50% (moderate stress conditions) was estimated at 85 μM and the higher concentration of the metal was set at a value double the first (i.e., 170 μM). Both selected concentrations of Cd were used in all immunocytochemistry, molecular, biochemical and ultrastructure experiments.

### Immunocytochemical Localization of Tubulin

Excised root fragments consisting of meristematic and elongation zones were immediately fixed for 3 h in a MT stabilizing buffer (MTSB: 50 mM Pipes pH 7.0, 5 mM MgSO_4_, 5 mM EGTA) containing 4% freshly prepared paraformaldehyde. The fragments were then dehydrated in a series of ethanol solutions and embedded in Steedman’s wax (Vitha et al., 2000). Samples were sectioned at a thickness of 7 μm with a rotary microtome. After dewaxing, sections were treated overnight at 4°C with primary anti-α-tubulin antibody (clone B-5-1-2, Sigma T5168) diluted 1:800 with PBS/1% BSA. Subsequently, the sections were rinsed six times in PBS and then incubated at 37°C for 2 h with FITC-conjugated anti-mouse secondary antibody (Sigma F5262) diluted 1:400 with PBS/1% BSA. After rinsing with PBS, the material was treated with a solution of propidium iodide (Sigma P4170) at a concentration of 1 μg/ml for 5 min, rinsed again and mounted in antifadent solution (Citifluor Ltd) on glass slides. Sections were observed with an LSM 510 confocal microscope (Carl Zeiss, Jena, Germany). On average, five different roots were investigated.

### RNA Isolation and Reverse Transcription

RNA isolation was carried out with the use of TriReagent (Sigma T9424) according to the manufacturer’s instructions. For reverse transcription, 1 μg RNA from each experimental variant was purified with the use of a Deoxyribonuclease Kit (Sigma AMPD1) and transcribed into cDNA using a ReverseTranscription Kit (Thermo Scientific Fermentas #K1622). For real-time PCR reactions, the obtained cDNA was diluted five times.

### Measurements of Gene Expression

The expression pattern was analyzed for seven genes encoding α-tubulin. The gene sequences for tubulins were derived from the Phytozome database^1^, and the primers, listed in **Table 1**, were designed using primer3 software^2^. Real-time PCR reactions were performed on a Rotor-Gene 6000 Thermocycler (Corbett) in 20 μl of reaction mixture containing 0.1 μM of each primer, 1 μl of diluted cDNA, 10 μl of Power SYBR Green PCR Master Mix (Applied Biosystems 4368577) and DEPC treated water. The real-time PCR reaction started with initial denaturation at 95°C for 5 min, followed by 45 cycles consisting of 10 s at 95°C, 20 s at 45°C and 30 s at 72°C and finalized by denaturation at a temperature rising from 72 to 95°C by one degree every 5 s. The Ct (cycle threshold) values were determined using a real-time PCR Miner (Zhao and Fernald, 2005), and relative gene expression was calculated according to the Pfaﬄ equation (Pfaﬄ, 2001) in relation to a reference gene – ubiquitin. Earlier studies showed that out of three potential tested reference genes (encoding ubiquitin, 18S rRNA, CDK-A), a *ubiquitin* gene exhibits the most stable expression in response to Cd and it has been used as reference gene in other studies concerning the impact of Cd on gene expression in soybean seedlings (Chmielowska-Bąk et al., 2013). Measurements were performed on samples from three independent experimental repetitions, with each sample consisting of a pool of at least 20 root tips (6 mm long).

**Table 1:** The names of analyzed genes, their accession numbers in Phytozome database and the designed primers.

Gene name	Number in Phytozome databse	Left primer	Right primer
*Tubα1*	Glyma04g09350	CCGAGTCTGGTGATGGAGAT	ACCACACATGTCCGACAGAA
*Tubα2*	Glyma05g23230	TGTCCTGCTCGACAATGAAG	GCACAAGGTTGGTCTGGAAT
*Tubα3*	Glyma06g09500	CTCCGTTGACTACGGGAAAA	CAACATCGGTGTGTTCAAGG
*Tubα4*	Glyma08g12140	TGAGGTGTTCTCTCGCATTG	AGCCCCAACCTCCTCATAGT
*Tubα5*	Glyma10g40150	CCAACCTCAACCGTCTTGTT	GGAGGAAAGCATGAAATGGA
*Tubα6*	Glyma17g16831	GTTTGATGGTGCATTGAACG	ACAACATCACCCCGGTACAT
*Tubα7*	Glyma20g27280	ATTGAGCGTCCCACCTACAC	GGAGGAAAGCATGAAATGGA

### Protein Extraction and 2D Gel Electrophoresis

Excised root tips (6 mm long) were ground in liquid nitrogen to a fine powder, and proteins were precipitated with cold TCA-2ME-acetone solution for at least 1 h at -20°C according to the procedure of Méchin et al. (2007). Protein solubilization was carried out with the use of DeStreak Rehydratation Solution (GE HealthCare), supplemented with 20 mM DTT and 0.2% Bio-Lyte buffer (BioRad). The protein concentration in the final samples was calculated using a commercial 2-D Quant Kit (GE HealthCare). The assay was executed according to the manufacturer’s instruction using BSA as a standard, and each sample was analyzed at least three times. Finally, approximately 100 μg of proteins were loaded onto 7 cm IPG strips with 4.7–5.9 pH gradient (BioRad). After overnight rehydratation, the strips were subjected to isoelectrofocusing (IEF) using Multiphor II (GE HealthCare) and a run was carried out as follows: 300 V (2 h), 1500 V (1.5 h), and 3500 V (8 h). After IEF separation, the strips were stored at -80°C. Prior to SDS-PAGE, the strips were equilibrated 2 × 15 min in an equilibration buffer (50 mM Tris-HCl, pH 8.8, 6 M urea, 30% glycerol, 2% SDS, 0.002% bromophenol blue), first containing 65 mM DTT, followed by an equilibration buffer with 135 mM iodoacetamide. For the second dimension separation, the strips were applied to 10% precast polyacrylamide gels (BioRad) and run in a Mini-PROTEAN Tetra Cell (BioRad) at a constant current (20 mA per gel) with a Prestained Protein Ladder (Thermo Scientific). After separation, the proteins were blotted onto PVDF membranes.

### Antibodies

The following antibodies were used in the detection of tubulin subunits: mouse monoclonal antibody B-5-1-2 (IgG1; Sigma T5168; diluted 1:5,000) was used to recognize an epitope located in the C-terminal end of the α-tubulin isoforms; mouse monoclonal antibody TU-01 (IgG1; Novus Biologicals NB500-333; diluted 1:2,000) was directed against the N-terminal structural domain (epitope aa 65–97) of the α-tubulin; and mouse monoclonal antibody TU-06 (IgM; Novus Biologicals NB120-7792; diluted 1:2,000) was directed against the N-terminal structural domain of β-tubulin and reacted with all charge variants of tubulin. Post-translationally modified tubulins were detected with the following antibodies: mouse monoclonal anti-tyrosine tubulin antibody TUB-1A2 (IgG3; Sigma T9028; diluted 1:5,000), which is non-reactive with cells that have been treated with pancreatic carboxypeptidase A under conditions which remove only the C-terminal tyrosine; monoclonal anti-acetylated antibody 6-11B-1 (IgG2b; Sigma T6793; diluted 1:2,000), which recognizes an epitope located on the α3 isoform of *Chlamydomonas* axonemal α-tubulin, within four residues of Lys-40 when this amino acid is acetylated; mouse monoclonal antibody GT335 (IgG1; Enzo Life Sciences ALX-804-885; diluted 1:2,000), which recognizes most forms of polyglutamylated tubulin and other polyglutamylated proteins, independent of the length of the glutamate side chains; and, finally, rabbit polyclonal antibody against detyrosinated tubulin (IgG; Millipore AB3201; diluted 1:1,000), which specifically recognizes the detyrosinated form of the tubulin α-chain (Glu tubulin). All used primary antibodies were highly specific against particular modifications of tubulins with an exception of GT335 which might recognize also other glutamylated proteins. Secondary anti-mouse or anti-rabbit antibodies conjugated with horseradish peroxidase (HRP) were provided by Agrisera (AS11 1772, diluted 1:20,000; AS09 602, diluted 1:30,000) or Santa Cruz Biotechnology (sc-2064, diluted 1:10,000).

### Protein Blotting and Immunostaining

Proteins were blotted onto PVDF membranes (Millipore) with the use of a TE22 Mighty Small Transfer Tank (Hoefer) in CAPS buffer (10 mM CAPS, 10% methanol, 0.01% SDS) for 1 h at a constant current of 1 mA per 1 cm^2^ of membrane (70 mA). The quality of transfer was evaluated by staining gels with CBB-R250 and checking the correct blotting of pre-stained molecular mass standards (Thermo Scientific). Membranes were stained with freshly prepared Ponceau S [0.1% Ponceau S (w/v) in 5% acetic acid] to verify equal protein loading, and then blocked overnight with 5% BSA in a TBST buffer (10 mM Tris pH 8.0, 150 mM NaCl, 0.05% Tween 20) followed by incubation with different primary antibodies diluted in a TBST buffer. After five extensive washes in the TBST buffer, the membranes were incubated with the appropriate secondary antibodies and after extensively washing with the TBST buffer, the immunological reaction was visualized by the use of Lumi-Light Western Blotting Substrate according to the manufacturer’s instructions (Roche). The chemiluminescent signal was captured on X-ray film (Fuji), and protein spots’ level of intensity was analyzed by means of MultiGauge (release 2.2) Fuji software. The quantitative results were calculated as a ratio of pixel intensity values to area of spots, and the data were presented considering control or 170 μM Cd as a reference point (100%). At least three independent blots from different experiments were analyzed. Some blots were stripped with a harsh stripping solution (62.5 mM Tris-HCl, pH 6.8, 2% SDS, 100 mM 2-ME) for 30 min at 70°C and reprobed again to check cross reactivity with other antibodies against tubulin applied in the study.

### Transmission Electron Microscopy

Excised root tip fragments (3 mm long) were immediately fixed in a mixture of 2% (v/v) paraformaldehyde and 2% (v/v) glutaraldehyde in 0.05 M cacodylate buffer, pH 6.8, for 3 h at room temperature. After three washes in a cacodylate buffer, 10 min per wash, cells were postfixed with 1% OsO_4_ in the same buffer overnight at 4°C. The roots were then dehydrated in a graded ethanol series with 1% uranyl acetate prestaining at the 70% alcohol step (overnight at room temperature). The material was embedded in LR white resin (Sigma 62662), and polymerized at 60°C for 24 h. The roots were cut into ultrathin sections (70 nm) using a Reichert Ultracut S (Leica, Austria) microtome. Sections for TEM were stained with an aqueous solution of 9% uranyl acetate followed by 0.5% lead citrate. The ultrastructure of cells was examined under a Jem 1200 EX II (Jeol Co., Japan) transmission electron microscope at 80 kV. Sections of three different roots were prepared and viewed.

## Results

### Root Growth Under Cadmium Stress

**FIGURE 1:**
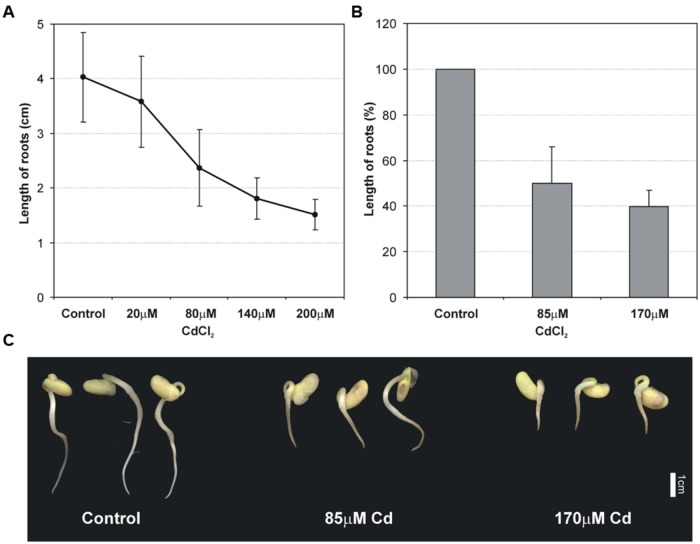
**Effect of different Cd concentrations on the primary root growth of 2 days old soybean seedlings (A)**. Effect of moderate (85 μM Cd) and high (170 μM Cd) cadmium stress on root length **(B)** and morphology of seedlings **(C)**. The results represent the mean with standard deviation of three independent experiments after 48 h treatment.

The effect of Cd on the root growth was examined 48 h after the incubation of seedlings in Cd solutions with Cd at concentrations of 20, 80, 140, and 200 μM. It was found that Cd limited root growth proportionally to the concentration of the metal in the solution (**Figure 1A**). The measurements allowed tolerance index to be calculated for root growth. Determined concentrations for moderate stress (85 μM) and high stress (170 μM) conditions were tested again in terms of root growth inhibition and a significant and gradual reduction in root growth was observed (**Figure 1B**). In addition to significant root growth inhibition, Cd also altered the morphology of roots, which became brownish (**Figure 1C**) and more brittle than the control roots.

### Cadmium Effects on Microtubule Organization in Root Cells

**FIGURE 2:**
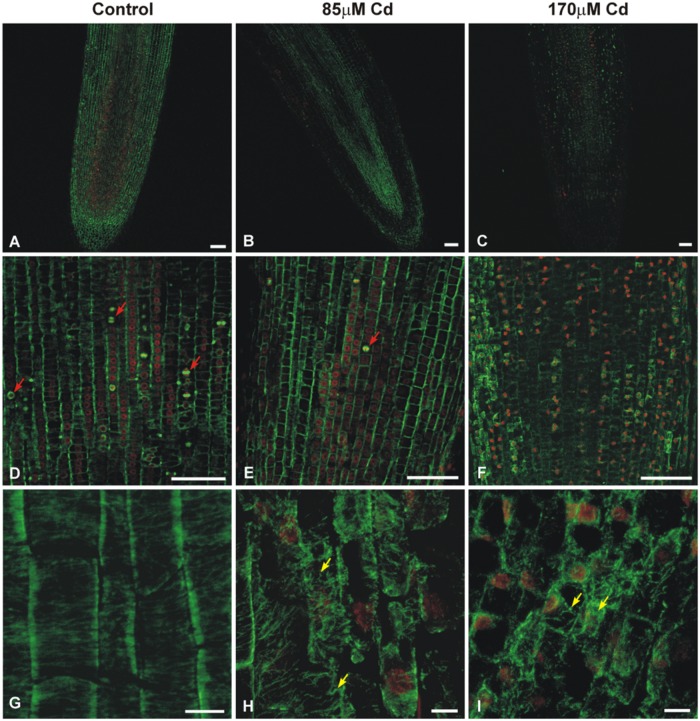
**Median longitudinal sections of soybean root apices treated with Cd indicating fluorescence signal strength, mitotic activity, and cortical MTs structure**. The sections were double-labeled with anti-α-tubulin antibody (B-5-1-2) and DNA-binding dye (propidium iodide). An overview of untreated control roots **(A,D,G)** and the roots of seedlings treated with 85 μM **(B,E,H)** or 170 μM Cd **(C,F,I)**. Exposure to Cd results in a differentiated MT immunofluorescence signal, a strong reduction in the number of proliferating cells (red arrows indicate cells in different stages of mitosis) and different stages of cortical MTs structure disorders in cells of cortex tissue (yellow arrows). Bar = 100 μm **(A–F)**, 10 μm **(G–I)**.

**FIGURE 3:**
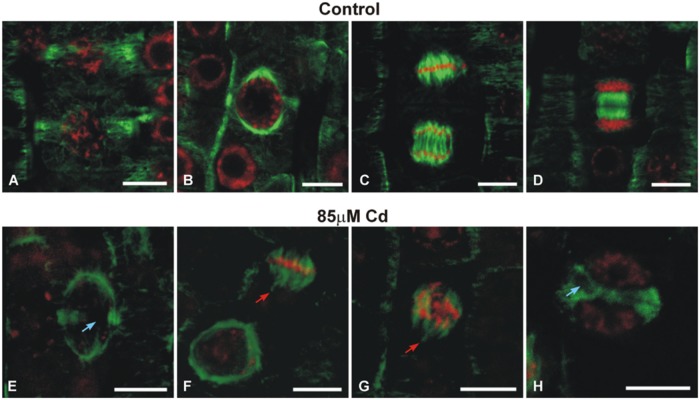
**Images showing the effects of moderate Cd (85 μM) treatment on the mitotic MT arrays of soybean root apical cells**. Merged images of immunofluorescence-labeled MTs and propidium iodide-stained nuclei. Cells of control roots displaying preprophase band **(A)**, perinuclear spindle **(B)**, mitotic spindle **(C)** and phragmoplast **(D)**. Different mitotic arrays disorders (arrows) under Cd stress **(E–H)**. Bar = 10 μm.

The double-label staining of longitudinal root tip sections with anti-α-tubulin antibody and DNA-binding dye (propidium iodide) revealed differentiated MT signal intensity (green immunofluorescence) under Cd treatment (**Figure 2**). The most pronounced fluorescence was observed in sections of control roots (**Figure 2A**). At a moderate concentration of the metal (85 μM), the main fluorescence signal was observed in procambium cells (**Figure 2B**), whereas at a high Cd treatment (170 μM), a faint staining was observed limited mostly to the procambium area (**Figure 2C**). Moreover, higher magnification of the control sections revealed many cells at different stages of division (**Figure 2D**), while under moderate Cd stress a significant reduction in mitotic activity (approximately 60%) was observed (**Figure 2E**). No cell divisions were observed in the root sections of seedlings treated with a high Cd concentration (**Figure 2F**). The highest magnification of control cells made it possible to observe a detailed picture of the stained MT cytoskeleton at different stages of the cell cycle, including cortical MTs (**Figures 2G–I**), preprophase band (**Figure 3A**) and perinuclear MTs (**Figure 3B**), mitotic spindle (**Figure 3C**) and phragmoplast (**Figure 3D**). The cortical MTs in control cells of soybean roots formed a fine, subtle and dense network orientated perpendicular to the long axis of the cells in the procambium and cortex area (**Figure 2G**). On the other hand, in the cells of seedlings treated with Cd the structure pattern of cortical MTs was significantly deformed in the cortex tissue, and included a decrease in the number of cortical MT bundles, randomization of the microtubular network, thickenings on individual MT fibers (dot-like staining) and discontinuous wavy MT bundles (**Figure 2H**). Finally, a complete disassembly and depolymerization of the MTs occurred, evidenced by short MT fragments and amorphous clusters of fluorescence signal, especially pronounced in the external cells of cortex tissue (**Figure 2I**). In contrast to the cortex area, the procambium cells of Cd-treated seedlings showed no or much less evident changes in their MT structures. The dividing cells observed at a moderate concentration of Cd also displayed a distorted structure of the MT cytoskeleton, such as arrays without MT bunches (**Figures 3E,H**) or with MT fibers outside the main body of the array (**Figures 3F,G**). In control experiments, neither the primary antibody B-5-1-2, nor the FITC-conjugated secondary antibody alone gave any specific staining.

### Expression of Genes Encoding α-tubulin

**FIGURE 4:**
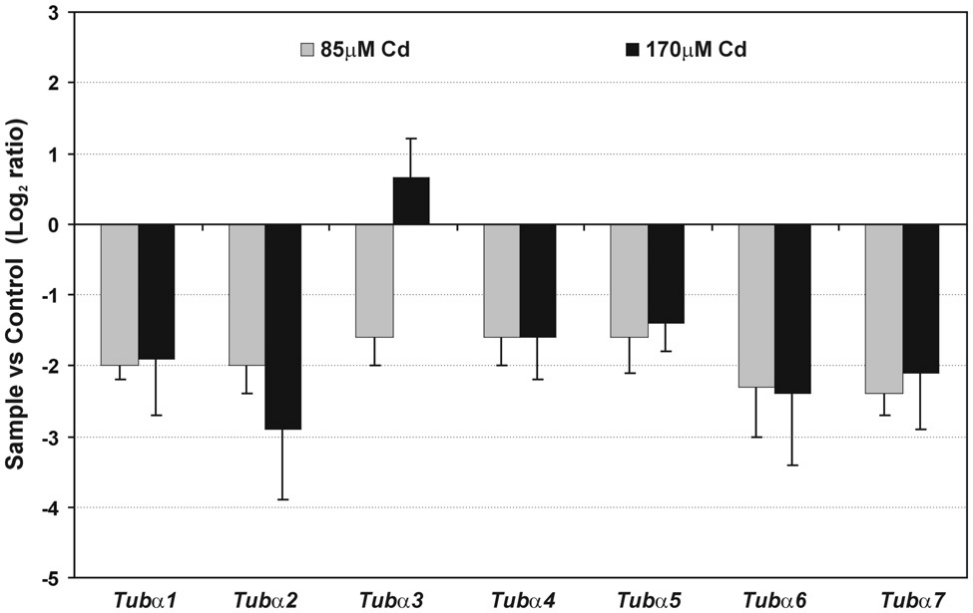
**Relative expression of gene encoding α-tubulin isotypes**. Fold change evaluated through real time PCR after moderate (85 μM Cd) and high (170 μM Cd) metal treatment. Values shown in the histogram are represented as a log_2_ fold change compared to the control sample average of 0 (untreated seedlings). The results represent the mean (+SE) of three separate experiments.

Cadmium treatment also had detrimental effects on the expression of the vast majority of analyzed genes encoding α-tubulin (**Figure 4**). A significant decrease (generally over 50%) in the expression of all estimated genes was observed in moderate (85 μM) and high (170 μM) Cd treatments. A weak increase in expression of one gene *Tubα3* was found at a high concentration of Cd compared to control seedlings. Moreover, in the case of three genes – *Tubα1, Tubα5, and Tubα7* – the decrease in genes expression was more pronounced in response to moderate than to high Cd concentration.

### Accumulation of Tubulin Isoforms Under Cadmium Stress

Protein extracts were separated by means of two-dimensional electrophoresis and after western blot, probed with a set of different monoclonal antibodies against tubulin. In the IEF dimension, a very narrow pH gradient (4.7–5.9) of IPG strips were used, and a second dimension was carried out with precast SDS gels in order to improve the resolution and reproducibility of the tubulin isoforms. No cross-reaction with other proteins was observed and the secondary antibody alone gave no immunostaining signal. Moreover, the used antibodies stained all typical MT arrays appearing in plant cells during the cell cycle, including cortical MT, mitotic spindle and phragmoplast.

**FIGURE 5:**
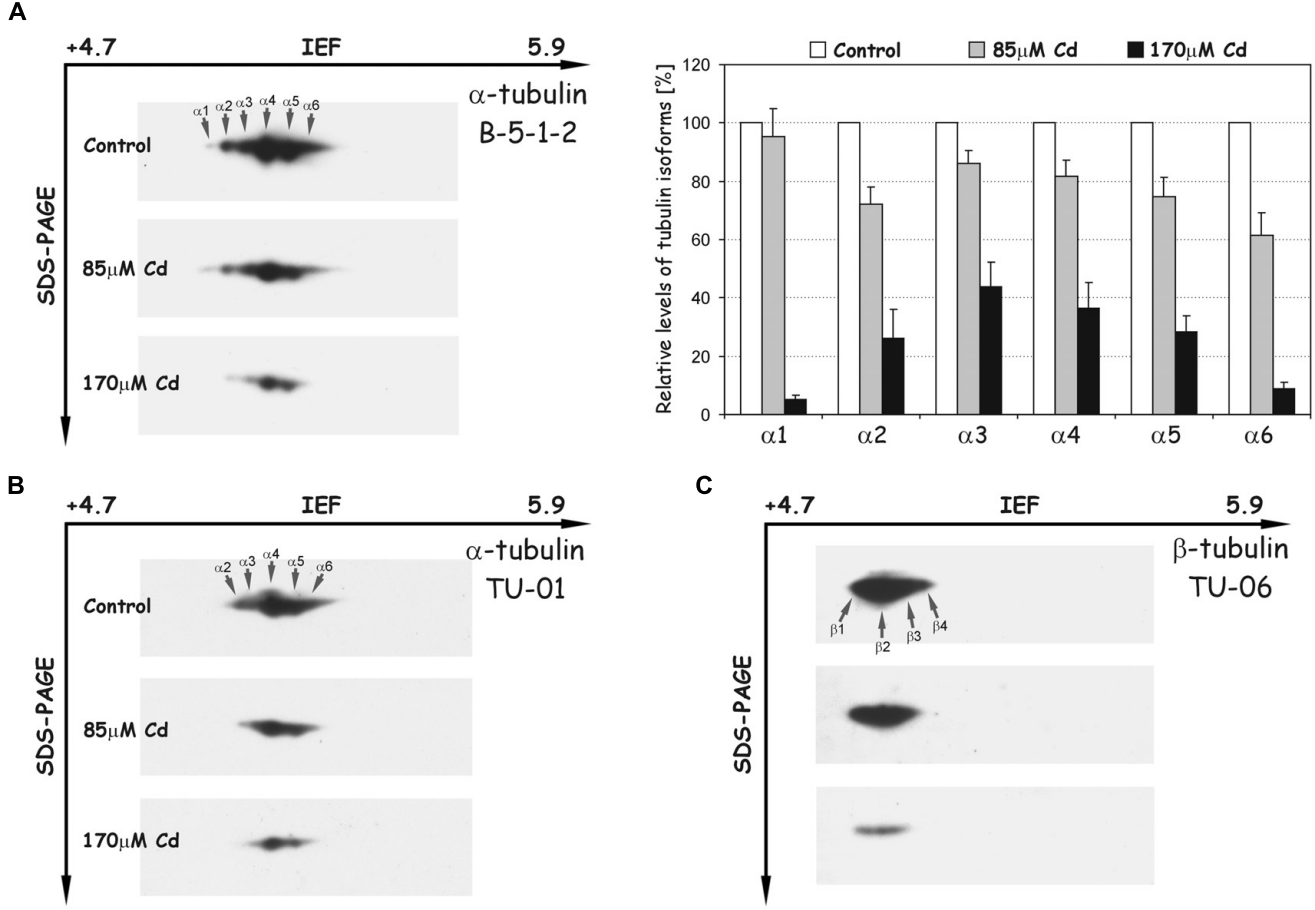
**Representative immunoblots probed with different antibodies against α-tubulin **(A,B)** and β-tubulin isoforms (C)**. Individual tubulin isoforms are denoted by arrows marked α1–α6 for α-tubulin and β1–β4 for β-tubulin. The quantitative results for α-tubulin (antibody B-5-1-2) were calculated as a ratio of pixel intensity values to area of spots and data were presented considering the control as a reference point (100%). The values represent the average of three independent measurements with a standard deviation.

The most abundant electrophoretic pattern was found in control roots examined with antibody B-5-1-2, where six different isoforms of α-tubulin were identified (α1–α6), which have been numbered according to their position from acidic to basic pH (**Figure 5A**). The immunodetected isotubulins possess an average molecular mass of around 50 kDa and differ in their isoelectric points (pIs) ranging from approximately 5.1–5.3. After Cd treatment, the level of identified spots progressively decreased, and under high stress (170 μM Cd) the less pronounced α1 and α6 isotubulins were hardly detectable. The observed tendencies were verified by immunoblotting with other specific antibodies against α-tubulin (TU-01, **Figure 5B**) and β-tubulin (TU-06, **Figure 5C**). The first antibody recognized five distinct isoforms of α-tubulin, which in terms of pIs and molecular masses corresponded to isotubulins α2, α3, α4, α5, and α6 detected with B-5-1-2 antibody. In turn, the anti-β-tubulin antibody immunostaining made it possible to visualize four isoforms (β1–β4), which were focused in a distinct single cluster. The β-isotubulins were slightly more acidic in the IEF first dimension, and their pI values were in the approximate range 5.0–5.2. Similar to B-5-1-2 and TU-01 antibodies, the accumulation level of β-tubulin isoforms, recognized with TU-06 antibody, decreased significantly after Cd treatment, especially at high concentration of the metal.

### Post-Translational Modifications of Tubulin Under Cadmium Stress

**FIGURE 6:**
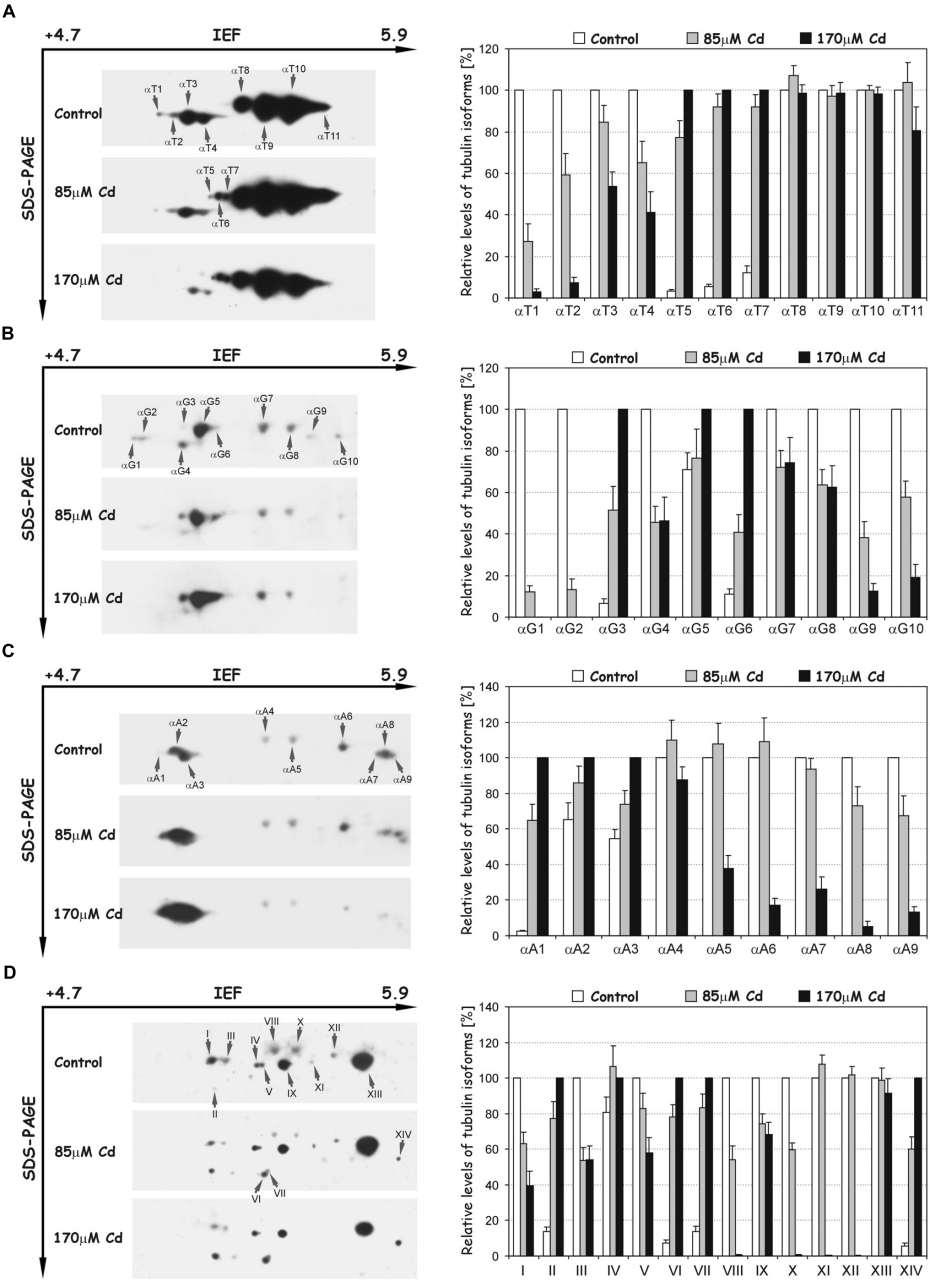
**Representative immunoblots probed with a set of different antibodies against tyrosinated α-tubulin **(A)**, detyrosinated α-tubulin **(B)**, acetylated α-tubulin **(C)** and polyglutamylated proteins (D)**. Individual tubulin isoforms (spots) are denoted by arrows marked: αT1–αT11 (tyrosinated isoforms), αG1–αG10 (detyrosinated isoforms), αA1–αA9 (acetylated isoforms) and I–XIV (polyglutamylated proteins). The quantitative results were calculated as a ratio of pixel intensity values to the area of spots, and the data were presented considering the control or 170 μM Cd as a reference point (100%). Values represent the average of three independent measurements with a standard deviation.

Post-translational modifications were examined with the set of different specific antibodies recognizing tyrosinated, detyrosinated, acetylated, and polyglutamylated isoforms of tubulin (**Figure 6**). The use of the antibody TUB-1A2, which recognizes a peptide containing the carboxy terminal amino acid tyrosine of α-tubulin, enabled detection of 11 isoforms (αT1–αT11, **Figure 6A**). The distinguished isoforms were focused in two distinct clusters of spots. The first cluster consisted of four more acidic isoforms (αT1–αT4) with a molecular mass of about 50 kDa and pIs at the range 5.1–5.3. The isoforms in the first cluster were identical in terms of molecular mass and pI values, with tubulin spots α2–α5 recognized with B-5-1-2 antibody. After Cd treatment, the intensity of the spots progressively decreased and under high stress (170 μM) the more acidic isoforms (αT1 and αT2) were hardly detectable (**Figure 6A**). The second cluster of spots (αT5–αT11) migrated more slowly in the SDS-PAGE second dimension than in the first one, and the tubulin isoforms were slightly less acidic. The most abundant isoforms αT8–αT11 were relatively stable after Cd treatment. In contrast, spots αT5–αT7, which were weakly recognized in the control roots, became more abundant in roots treated with the metal (**Figure 6A**).

A differential signal was also detected in the recognition of detyrosinated isoforms of α-tubulin (Glu-tubulin), where a population of 10 distinct spots (αG1–αG10) was distinguished (**Figure 6B**). The molecular mass of spots was around 50 kDa, but their pI values extended over the range of approximately 5.1–5.5. The relative level of most recognized spots significantly decreased in comparison to the control roots, especially under high Cd stress (170 μM), where some spots (αG1, αG2) were not detectable. On the other hand, the accumulation of three tubulin isoforms: αG3, αG5, and αG6 significantly increased in Cd-treated roots (**Figure 6B**). Additionally, spots αG4, αG7, and αG8 corresponded in terms of molecular mass and pI values to isoforms α4 (**Figure 5A**), αT9 and αT10 (**Figure 6A**), recognized with B-5-1-2 or TUB-1A2 antibodies, respectively.

To identify the isoforms post-translationally modified by acetylation, the antibody 6-11B-1 was used, and nine spots of acetylated α-tubulins (αA1–αA9) were recognized (**Figure 6C**). The molecular masses of spots were approximately 50 kDa and their pI values extended between 5.1 and 5.9. The level of less-pronounced spots αA4–αA9 significantly decreased under high Cd stress compared to control and moderate stress (85 μM Cd) levels. A quite opposite situation was observed in the case of more acidic and more pronounced isoforms αA2 and αA3, where a progressive increase in their accumulation occurred (**Figure 6C**). Moreover, spots αA3, αA4, αA5, and αA6 were identical in regard to their pI values and molecular masses with isoforms α4 (**Figure 5A**), αT9, αT10 (**Figure 6A**) and αG10 (**Figure 6B**), identified with B-5-1-2, TUB-1A2 and anti-Glu-tubulin (6-11B-1) antibodies, respectively.

In the next set of experiments the polyglutamylation of proteins with GT335 antibody was examined. A pattern of 14 polypeptides (I-XIV) with glutamate side chains was recognized (**Figure 6D**). The isoelectric points and molecular masses of the distinguished spots were in the range of 5.2–5.7 and 45–50 kDa, respectively. The level of detected peptides under Cd stress changed differentially. On the one hand, the accumulation of polypeptides I, III, V, VIII, IX, X, and XII progressively decreased under moderate and high stress (**Figure 6D**). On the other hand, the level of spots II, IV, VI, VII, and XIV distinctively increased after Cd treatment. Moreover, the polypeptides I, III, VIII, X, and XII were identical in terms of pI values and molecular masses with tubulin isoforms α4, α5 (**Figure 5A**), αT9, αT10 (**Figure 6A**) and αA6 (**Figure 6C**), respectively.

### Cadmium Effects on the Ultrastructure of Root Cells

**FIGURE 7:**
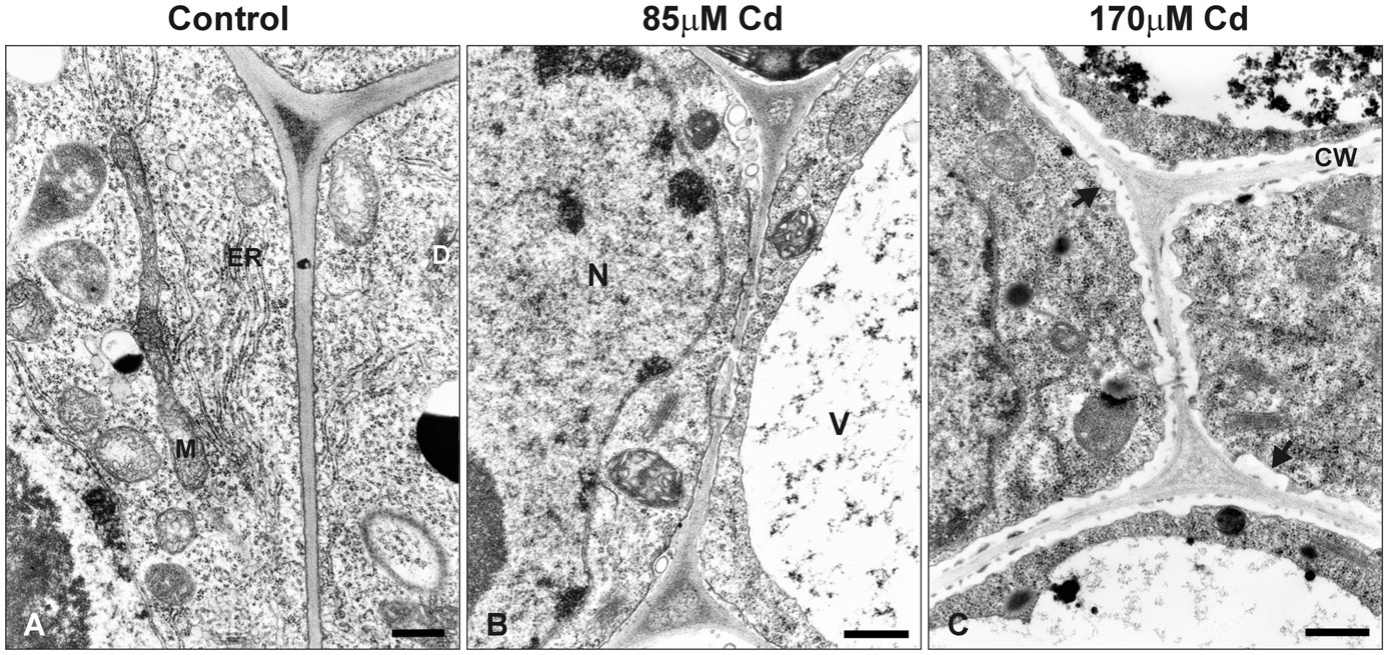
**Electron micrographs of the root tip cells of control seedlings **(A)** and plants treated with moderate **(B)** or high **(C)** Cd concentrations (85 or 170 μM Cd, respectively)**. M, mitochondrion; N, nucleus; V, vacuole; ER, endoplasmic reticulum; D, dictyosome; CW, cell wall. Black arrows indicate irregular deposition of callose in the root cells of Cd-treated seedlings. Bar = 500 nm.

At the ultrastructural level, the cells of control roots displayed thin cell walls and dense cytoplasm filled with numerous organelles and structures including mitochondria, endoplasmic reticulum, and dictyosomes (**Figure 7A**). In the presence of Cd, distinct changes in the ultrastructure were observed, the most prominent of which included development of one central located vacuole (**Figure 7B**) and irregular deposition of callose, especially in high Cd treatment (**Figure 7C**).

## Discussion

The plant MT cytoskeleton promptly rebuilds its arrangement in response to various intracellular and external stimuli, including abiotic stresses (Smertenko et al., 1997c; Schwarzerová et al., 2002; Shoji et al., 2006; Lü et al., 2007; Liu et al., 2009; Xu et al., 2009). The results presented in this study confirmed that both root growth and structure of MTs are very sensitive to Cd treatment. Previous studies performed in our department revealed that soybean seedlings respond to Cd in the range of the applied concentrations differentially in the terms of antioxidant response (Pawlak et al., 2009), activation of phenylpropanoid pathway (Pawlak-Sprada et al., 2011) and expression of signaling associated genes (Chmielowska-Bąk et al., 2013). Moreover, this study demonstrates that root tips in high Cd stress (170 μM), contrary to moderate stress (85 μM), were distinguished by the lack of dividing cells (**Figure 2**) and more advanced callose deposition (**Figure 7C**). It can be concluded that increasing concentrations of the metal and resulting reduction in root growth were strongly correlated with a drop in the number of mitotic cells and an increase in abnormalities in MT structure (**Figures 2** and **3**). A similar pattern of MT dysfunction under short-term Cd treatment (0.5–24 h) was observed in the root tips of garlic (Xu et al., 2009), pea (Fusconi et al., 2007), and onion (Dovgalyuk et al., 2003) treated with very low (0.25 or 10 μM) to relatively high concentrations of the metal (50–250 μM). A significant reduction in the number of mitotic cells is in line with other studies showing that exposure to Cd arrests the entrance of the cells into a division cycle (Fusconi et al., 2006; Amirthalingam et al., 2013; Arya and Mukherjee, 2014; Shi et al., 2014). Thus, it seems to be well established that regardless of the plant species or experimental setup, a general feature of Cd toxicity is the rapid and sensitive response of the MT cytoskeleton connected with a decrease in the mitotic index. As yet, the exact mechanism of noxious Cd action toward MTs is not well understood. There are a few possibilities which include indirect Cd influence on MTs functioning by effecting the expression of tubulin genes, PTMs of the expressed proteins, and MAPs dysfunction. On the other hand, the direct interaction of the metal with the sulfhydryl groups of MTs, which are essential for MT polymerization, cannot be excluded (Wallin and Hartley-Asp, 1993).

In our study 7 genes for α-tubulin were examined. Most of the examined genes were down regulated after Cd treatment with the exception of *Tubα3*, which under high metal stress was slightly up-regulated (**Figure 4**). It is assumed that each plant species has evolved diverse sets of tubulin genes, which generate significant heterogeneity in the number of α- and β-tubulin isotypes. Consequently, isotypes of tubulin are differentially expressed in relation to the specific organ/tissue, developmental stage or changed environmental conditions (Breviario et al., 2013). Thus, in the context of the high heterogeneity of plant tubulin genes, one cannot exclude the participation of particular isotypes in specific processes. There are, for example, several premises that specific tubulin isotypes are associated with the synthesis of secondary cell wall layers. In *Eucalyptus grandis* expression pattern of specific β-tubulin gene was correlated with the deposition of the cell wall (Spokevicius et al., 2007). Moreover, in *Populus* increase in cellulose synthesis was accompanied by elevated expression of some β-tubulin isotypes (Oakley et al., 2007). In the present study, ultrastructure analysis of soybean root cells revealed that after high Cd treatment (170 μM), a noticeable deposition of callose occurred (**Figure 7C**), which may be considered as a cellular defense reaction by modification of cell wall properties to prevent metal uptake or enable its immobilization in cell walls (Krzesłowska, 2011; Eleftheriou et al., 2012; Piršelová et al., 2012). This result together with the observed induction of *Tubα3* gene in response to high Cd concentration might suggest that expression of at least some tubulin genes is also process-specific – induction of specific isotypes might be advantageous in stress conditions (Breviario and Nick, 2000). However, the majority of examined genes were significantly down-regulated after Cd treatment (**Figure 4**), but there was no differences in transcript level between moderate and high Cd concentrations. At the same time, the western blot analyses demonstrated reduction in the accumulation of tubulin isoforms in the concentration dependent manner (**Figure 5**). The observed discrepancy might result from modulation of various post-transcriptional and post-translational mechanisms (Maier et al., 2009; Vélez-Bermúdez and Schmidt, 2014). For example, translation efficiency might be impaired by miRNAs, changes in mRNA structure or hampered assembly of ribosome-mRNA complexes. Additionally, it can not be excluded that different Cd concentrations might have distinct impact on the lifetime of synthesized proteins.

The disturbance observed in MTs functioning was accompanied not only by lower accumulations of α- and β-tubulin isoforms, but also by significant changes in the level of their PTMs. In our experimental approach we used the set of commercial antibodies against PTMs, which have been used successfully in other plant species (Smertenko et al., 1997a; Wang et al., 2004; Parrotta et al., 2010; Nakagawa et al., 2013). Based on animal and human cell models it is well documented that PTMs can modify interactions with MAPs (Verhey and Gaertig, 2007). A good example are plus-end tracking proteins (+TIPs), a highly diverse group of MAPs, which help control MT interactions by dynamic accumulation at the distal, highly tyrosinated ends of growing MTs (Akhmanova and Hoogenraad, 2005; Hammond et al., 2008). In this way, tyrosination of MTs guides +TIPs localization and influences their function. It is assumed that stable MTs exhibiting less dynamic behavior are enriched in detyrosinated tubulin subunits, while dynamic MTs include tyrosinated tubulin subunits (Webster et al., 1987; Bulinski and Gundersen, 1991). The results presented in this paper imply that Cd contributes to stabilization of the MTs because the level of a quite large part of tyrosinated tubulin isoforms (e.g., αT1–αT4) significantly decreased in a metal-concentration dependent manner (**Figure 6A**). Moreover, the tyrosinated isoforms (αT1–αT4) were also identical in terms of their pIs and molecular masses to down-regulated tubulin isoforms (α2–α5) recognized with B-5-1-2 and TU-01 antibodies (**Figure 5**). Simultaneously, the general level of the most pronouncedly detyrosinated isoforms increased (**Figure 6B**); however, the pattern of this modification was not unequivocal. Although, detyrosination of one of the α-tubulin isoform (αG4) was markedly diminished under stress conditions, at the same time, a significant increase of other isoforms (αG3, αG5, and αG6) occurred.

A similar situation was observed in the case of acetylated isoforms, where the most pronounced isotubulins αA2 and αA3 significantly increased under stress conditions (**Figure 6C**). Acetylation of tubulin is mainly considered a marker of stable MTs resistant to turnover (Webster and Borisy, 1989), albeit tubulin acetylation has been also discovered on dynamic MTs (Perdiz et al., 2011). The function of acetylated MTs still remains unclear, and most suggestions concern human cells functioning. It is proposed that acetylation, similar to tyrosination/detyrosination modifications, can influence the transport and binding of MAPs to selected MTs (Gardiner et al., 2007). However, most interactions between MAPs and MTs take place on the outer surface of MTs, and it remains a mystery how acetylated tubulin serves as a guide for MAPs because the primary site of tubulin acetylation Lys40 is orientated toward the MT lumen. However, the use of a proteomic approach allowed recognition of some new sites of tubulin acetylation, some of which are exposed on the outer surface of MTs (Choudhary et al., 2009). Therefore, a plant-specific site of tubulin acetylation on the outer surface of the MT can also not be excluded. A similar pattern in tubulin PTMs was observed in the sponge *Clathrina clathrus*, where exposure to a sublethal concentration of Cd for 24 h reduced the level of tyrosinated α-tubulin and simultaneously enhanced the level of detyrosinated and acetylated α-tubulin (Ledda et al., 2013). The predominance of our study is the higher resolution of tubulin electrophoretic separation due to the application of 2D technique enabling gaining insight into the population of particular tubulin isoforms. We have shown that among the tubulin isoforms, in some the level of modifications increased (e.g., αT5–αT7), but at the same time, the other detected isotubulins exhibited a lower level of modifications compared to control variants (e.g., αT1–αT4). The obtained results draw attention to the fact that apart from the general level of PTMs, their great significance under stress or altered environmental conditions might be associated with increased modification of particular tubulin isoforms, which in turn might be derived from specific isotypes of tubulin. Nonetheless, it has been shown that evolutionarily distant organisms such as sponge and soybean exhibited a similar pattern of response to Cd stress. The over-accumulation of detyrosinated and acetylated α-tubulins with a concurrent depletion of tyrosinated tubulin might be a general mechanism involved in maintaining the efficient structural and functional integrity of the MT cytoskeleton under Cd stress. This assumption might be strengthened by the observations of MT cytoskeleton functioning under other heavy metal stress like hexavalent chromium. The studies of different *Fabaceae* species indicate on MTs stabilization by increased acetylation of α-tubulin in a time- and concentration-dependent manner under chromium stress (Eleftheriou et al., 2013, 2015).

The putative increase in stability of the MT structure after Cd treatment seems to be in line with changes observed in the accumulation of polyglutamylated isoforms. In our study we recognized a population of 14 spots which were modified by the addition of one or more glutamate residues, but only five of them (I, III, VIII, X, and XII) have identical properties in terms of pIs and molecular masses with tubulin isoforms recognized with B-5-1-2, 6-11B-1, and TUB-1A2 antibodies (**Figures 5** and **6**). However, one should bear in mind that the antibody GT335, conversely to other antibodies applied here, might recognize some other proteins modified by glutamylation. Thus, the rest of the spots might not be related to tubulin proteins or, due to the right pI and molecular masses, might be associated, in at least some cases, with β-tubulin isoforms or γ-tubulin proteins. In our study the highest levels of polyglutamylated tubulin isoforms were recognized in control roots, and significantly decreased after Cd treatment, especially at high concentration of the metal, where some of them (VIII and X) were almost undetectable (**Figure 6D**). The exact function and significance of tubulin polyglutamylation is elusive and hypothetical, especially in plant cells. However, studies on human cells demonstrated that glutamylation, and in particular the generation of long glutamate side chains, promotes the severing of MTs and in this way provides a novel mechanism for controlling the mass and stability of MTs (Lacroix et al., 2010). By analogy, if the same mechanism is functioning in plant cells, the decreased level of polyglutamylated isotubulins revealed in Cd-treated seedlings would restrict the MT-severing activity and prevent disassembles of stable MTs. This would be consistent with the results obtained with the use of antibodies against detyrosinated and acetylated isoforms of tubulin, which seems to indicate the increased stability of MTs after Cd treatment.

Taken together, the results obtained in this study confirmed that the MT cytoskeleton is one of the cellular structures most sensitive to Cd stress. The vast majority of examined α-tubulin genes together with most of recognized α- and β-tubulin isoforms were down-regulated. However, for the first time in plants, changes in PTMs level of tubulin isoforms after Cd treatment were demonstrated. The immunological approach with the use of a set of antibodies against different PTMs of tubulin imply the increased stability and reduced turnover of MTs during stress conditions. The stability of MTs in Cd-treated seedlings might be associated with a general increase in the levels of some detyrosinated and acetylated isoforms. On the other hand, the level of some tyrosinated and polyglutamylated tubulin isoforms, which are assumed to favor the dynamic turnover of MT fibers, decreased. A shift in MT dynamics to more stable MT fibers would enable preferential transport and cell wall modification, e.g., callose deposition, which prevent Cd uptake or enable its immobilization in cell walls. The stable MTs enable the molecular motors to reach their destination, avoiding an unfavorable situation in which the MT track falls apart.

## Author Contributions

JG, RP, EG conceived and designed research. JG carried out immunocytochemistry, ultrastructure, 2D electrophoresis and western blot experiments, JC-B conducted real-time PCR experiments. All authors analyzed the data. JG wrote the manuscript. All authors read and approved the manuscript.

## Conflict of Interest Statement

The authors declare that the research was conducted in the absence of any commercial or financial relationships that could be construed as a potential conflict of interest.

## Footnotes

1 http://www.phytozome.net

2 http://bioinfo.ut.ee/primer3-0.4.0
